# Bacterial Degradation of Low-Density Polyethylene Preferentially Targets the Amorphous Regions of the Polymer

**DOI:** 10.3390/polym16202865

**Published:** 2024-10-10

**Authors:** Trinh Nguyen, Jan Merna, Everett Kysor, Olaf Kohlmann, David Bernard Levin

**Affiliations:** 1 Department of Biosystems Engineering, University of Manitoba, Winnipeg, MB R3T 5V6, Canada; umngu278@myumanitoba.ca; 2 Department of Polymers, University of Chemistry and Technology, 160 00 Prague, Czech Republic; jan.merna@vscht.cz; 3 LexMar Global Inc., Haverhill, MA 01835, USA; ekysor@lexmarglobal.com (E.K.); okohlmann@lexmarglobal.com (O.K.)

**Keywords:** polymer structure, low-density polyethylene, LDPE, biodegradation, microbial degradation

## Abstract

Low-density polyethylene (LDPE) is among the most abundant synthetic plastics in the world, contributing significantly to the plastic waste accumulation problem. A variety of microorganisms, such as *Cupriavidus necator* H16, *Pseudomonas putida* LS46, and *Pseudomonas chlororaphis* PA2361, can form biofilms on the surface of LDPE polymers and cause damage to the exterior structure. However, the damage is not extensive and complete degradation has not been achieved. The changes in polymer structure were analyzed using Time-domain Nuclear Magnetic Resonance (TD-NMR), High-Temperature Size-Exclusion Chromatography (HT-SEC), Differential Scanning Calorimetry (DSC), and Gas Chromatography with a Flame Ionization Detector (GC-FID). Limited degradation of the LDPE powder was seen in the first 30 days of incubation with the bacteria. Degradation can be seen in the LDPE weight loss percentage, LDPE degradation products in the supernatant, and the decrease in the percentage of amorphous regions (from >47% to 40%). The changes in weight-average molar mass (Mw), number-average molar mass (Mn), and the dispersity ratio (Đ) indicate that the low-molar mass fractions of the LDPE were preferentially degraded. The results here confirmed that LDPE degradation is heavily dependent on the presence of amorphous content and that only the amorphous content was degraded via bacterial enzymatic action.

## 1. Introduction

Synthetic plastics are ubiquitous in our society. Plastic production is estimated to increase from 450 million metric tons to almost 600 million metric tons between 2025 to 2050 [1]. The most abundant and widely used plastics include Polyethylene (PE), Polypropylene (PP), Polyvinyl chloride (PVC), Polyethylene terephthalate (PET), and Polystyrene (PS) [2]. These types of plastics make up products from different industries, such as packaging, construction, textiles, automotive, electronic, and pharmaceutical. Specifically, PE is forecasted to account for approximately 25% of global plastic production in 2025. Of the predicted 445 million metric tons of global thermoplastic production, 120 million metric tons will be PE. By 2030, PE will reach approximately 30% of global thermoplastic production, with an estimated 135 million metric tons of PE out of 480 million metric tons of global plastic [3,4]. Since plastics can be easily produced at low price, and the cost to collect and sort the used plastic is greater than de novo synthesis, production of new plastics is preferred over recycling [5]. The synthetic polymers used to manufacture plastic materials are highly recalcitrant to natural biodegradation processes, and thus accumulate in the environment, creating a global plastic pollution problem [6,7].

Polyethylene consists of long chain hydrocarbons with different degrees of branching, and is classified as high-density, low-density, or linear low-density PE, depending on the amount of branching. In high-density polyethylene (HDPE), there is minimal branching and thus, the hydrocarbon chains can be packed tightly together and form highly crystalline material. In low-density polyethylene (LDPE), the degree of branching is higher, preventing tight packing of the LDPE chains and thus, LDPE has lower crystallinity. Due to the high crystallinity degree, HDPE is more opaque, harder, has higher tensile strength and higher melting temperatures than other forms of PE [8]. Less crystalline LDPE has a slightly cloudy appearance and is more flexible than HDPE, with lower tensile strength and a lower melting temperature [9]. Both types of PE are thermoplastic, which means they can be repeatedly pliable or moldable at increased temperatures and keep the shape once they cool down [10]. PE is an ideal polymer for a wide range of products, including plastic bags, packaging films, pipes and tubes, medical equipment, and other disposable single-use plastic products, which are discarded and then can accumulate in landfills or the oceans, contributing to the plastic accumulation problem worldwide.

Plastic biodegradation is a complex process that involves both abiotic and biotic processes. The major abiotic factor in plastic degradation is UV irradiation from sunlight [11], while the major biotic factors in plastic degradation are bacteria and fungi. Biodegradation is a multi-step process that includes biodeterioration, bio-fragmentation, bioassimilation, and mineralization [12]. Biodeterioration results from colonization of the plastic surface and formation of a biofilm by microorganisms. The microbes secrete enzymes that chemically alter the structure of the polymer, resulting in changes in the surface of the material that can be observed by Scanning Electron Microscopy (SEM). The polymer surface may appear rough instead of smooth, and may have cracks or holes [13]. The next step in the biodegradation process is biofragmentation. Enzymes secreted by microorganisms cleave the polymer chains into smaller fragments, which may undergo bioassimilation and be used by microbes as carbon sources for metabolism and growth [12].

An increasing number of microorganisms, both bacteria and fungi, have been shown to deteriorate LDPE. Recently, *Cupriavidus necator* H16, *Pseudomonas putida* LS46, and *Pseudomonas chlororaphis* PA2361 were found to grow on minimal media with LDPE supplied as the main carbon source [14]. The average weight loss of LDPE incubated with each of the three bacteria over 21 days was reported at approximately 30% [14]. The ability to degrade such a high amount of LDPE in a short period of time makes these bacteria promising candidates for LDPE degradation research. Thus, they are used as the biotic agent in this study to see their effect on the LDPE.

Why LDPE is so resistant to biodegradation, and why these bacteria cannot completely degrade LDPE, is not well understood. It is well known that abiotic factors, such as UV light or oxidizing agents, can oxidize carbon–carbon bonds in the polymer chains, creating carbonyl groups that make the polymer more prone to degradation [15,16]. Oxidative pre-treatment of LDPE, or modification of LDPE to contain pro-oxidants, results in a higher degradation rate via bacteria compared to non-treated LDPE [17,18]. Degradation was concluded to occur primarily at the short-chain oligomers of the material [19]. However, complete degradation of LDPE has never been reported, especially after exposure to abiotic factors alone.

LDPE essentially consists of loosely branched (approx. 2%) alkane hydrocarbons, which contain sufficiently long, linear hydrocarbon chain segments that cannot be packed tightly, providing varying degrees of crystalline and amorphous regions [20]. The tightly packed crystalline regions may prevent access for microbial enzymes, limiting their ability to react with polymer chains. Structurally, the amorphous regions have less steric hindrance and may be more accessible to bacterial enzymes. Therefore, the degradation efficiency may depend on the percentage of amorphous versus the percentage of crystalline regions of LDPE structure. We investigated this hypothesis using various methods to detect changes in the structure of LDPE incubated with bacteria over different periods of time. Changes in the amorphous regions of LDPE structure were detected by Time-domain Nuclear Magnetic Resonance (TD-NMR). High-Temperature Size-Exclusion Chromatography (HT-SEC) was used for monitoring chain scission, while Differential Scanning Calorimetry (DSC) was used to measure the crystallinity content of the LDPE before and after bacterial treatment.

## 2. Materials and Methods

### 2.1. Bacteria Strain, Culture Media, and Growing Conditions

Three bacteria strains were used in this study: *Cupriavidus necator* H16, *Pseudomonas putida* LS46, and *Pseudomonas chlororaphis* PA2361. *C. necator* H16 was obtained from ATCC while *P. putida* LS46 was isolated from hog barn wastewater [21]. *P. chlororaphis* PA2361 derived from *P. chlororaphis* PA23 with a mutation in the *phzR* gene (PA23phzR) [22].

The bacteria were grown in minimal PE medium with the composition as follow: 5 g of Na_2_HPO_4_, 2 g of KH_2_PO_4_, 3 g of (NH_4_)_2_SO_4_, 0.15 g of KCl, 0.5 g of NaCl, 1.2 mg of CaCl_2_, 20 mg of MgSO_4_, 1 mg of Fe(III)NH_4_ citrate, and 1 mL of trace elements solution for 1 L of medium. Each liter of trace elements solution contains: 0.3 g of H_3_BO_3_, 0.2 g of CoCl_2_·6H_2_O, 0.1 g of ZnSO_4_·7H_2_O, 30 mg of MnCl_2_·4H_2_O, 30 mg of NaMoO_4_·2H_2_O, 20 mg of NiCl_2_·6H_2_O, and 10 mg of CuSO_4_·5H_2_O. There was no other carbon source provided besides LDPE powder. The LDPE powder was purchased from Millipore Sigma (MilliporeSigma Canada Ltd., Oakville, ON, USA, CAS #9002-88-4) with the weight average molecular weight M_w_ of ~4000 g/mol and the number average molecular weight M_n_ of ~1700 g/mol. Prior feeding to the bacteria, the LDPE was washed with 70% ethanol to remove surface impurities then air dried, until completely dry. LDPE (1% *w*/*v*) was supplied to each bacterial culture as the sole carbon source. Bacteria grown in minimal media without LDPE served as control. The bacteria flasks were cultured in continuous shaking at 150 rpm at 30 °C. The growth curves for each bacteria strain were generated based on the total fragmented protein in 1 mL of culture collected at different time points. Total protein quantification was performed following the Quick Start^TM^ Bradford Protein Assay (Bio-Rad Laboratories Canada Ltd., Missisauga, ON, USA, #5000201) kit.

### 2.2. Detection of LDPE Degradation Products

Samples of the supernatant from each bacterial culture were collected on days 2, 15, 21, 30, and 60 to determine the presence of LDPE degradation products. The GC analyses were performed on an Agilent 7890A GC (Agilent Technologies Canada Inc., Missisauga, ON, USA), split/splitless inlet (operated in split mode, split ratio 10:1), a DB23 capillary column (Agilent, 30 m × 250 µm × 0.25 µm), and an FID as a detector. Benzoic acid (1 mg/mL), added as an internal control, was methylated using equal volume of 15% sulfuric acid in methanol solution. The reaction was incubated at 96 °C in a water bath for 3 h, followed by the addition of 1 mL of water to stop the reaction. A standard of C7–C40 linear alkanes (MilliporeSigma Canada Ltd., Oakville, ON, USA, CAS #49452-U) was run on the GC-FID with the samples and used to identify degradation products. The supernatants from minimal media and bacteria with LDPE were used as control.

### 2.3. LDPE Weight Loss Calculation

The LDPE powder was then collected at different time points via gravitational filtration method for analysis. LDPE powder was filtered using single-fold paper towels (Kimberly-Clark Professional ^TM^, Thermo Fisher Scientific Inc., Winnipeg, MB, CA, USA), and then washed with PBS buffer and water to remove bacteria residue. The powder was then dried in the oven at 60 °C till constant weight was achieved. The % weight loss of LPDE was calculated using the following formula:

(1) $$\%WL=\frac{m_{i}-m_{f}}{m_{i}}\times 100\%$$

WL = percent weight loss of LDPE (%);

m_i_ = LDPE weight before bacterial treatment (g);

m_f_ = LDPE weight after bacterial treatment (g).

### 2.4. Structural Changes in LDPE after Incubation with the Bacteria

Structural changes to LDPE polymers used as substrate by the bacteria were evaluated by different methods. The molar masses and dispersity values of LDPE samples were determined by High Temperature-Size Exclusion Chromatography (HT-SEC/GPC). Thermal properties and the percentage of crystallinity were determined by Differential Scanning Calorimetry (DSC). Changes in the percentage of crystalline and amorphous regions of the residual LDPE were determined using Time-Domain Nuclear Magnetic Resonance (TD-NMR). The LDPE powder that was not treated with bacteria served as a control and is referred to as the “Day 0” sample.

#### 2.4.1. High Temperature—Size Exclusion Chromatography

After incubation of each bacterial culture with LDPE as the sole carbon source for 2, 15, 21, 30, 60, 75, and 95 days, samples of residual LDPE were collected, and the molar masses and dispersity values of the LDPE samples were determined by HT-SEC (Polymer Char, Valencia, Spain) equipped with infrared and viscometric detectors. About 1.5 mg of the LDPE samples was dissolved in 1 mL of 1,2,4-trichlorobenzene at 160 °C for 1 h with shaking. Next, 200 µL of each sample was run through two sets of PSS Polefin size exclusion columns (Linear XL, 10 µm, 7.8 × 300 mm + guard column) (Polymer standard service, Mainz, Germany) at 150 °C in 1,2,4-trichlorobenzene, stabilized with 300 ppm of Santonox R, at an elution rate of 1 mL/min. The software GPC One (Polymer Char) was used to evaluate the data using a relative calibration based on narrow dispersity Polystyrene (PS) standards (580–123,300 g/mol, Polymer Laboratories, Darmstadt, Germany) with correlation by Mark–Houwink constants of PS and linear PE. The LDPE powder that was not treated with bacteria served as a control, and is labelled as the “Day 0” sample.

#### 2.4.2. Differential Scanning Calorimetry

Samples of residual LDPE were collected after 2, 15, 21, 30, and 60 days of incubation with each bacterial culture and subjected to DSC. Polymer measurements were performed on a TA Instrument Discovery DSC250 Auto calorimeter (TA Instruments, New Castle, DE, USA). Temperature ranged from 40 °C to 170 °C at a rate of 10 °C/min. For both heating and cooling, the N_2_ flow was 50 mL/min. Melting temperatures and enthalpies of fusion were obtained from the second heating run. The percentage of polymer crystallinity was calculated from the area of melting endotherm, using the hypothetical 100% crystallinity value of polyethylene, which is 293 J/g [23], based on the following equation, where dH_m_ is the normalized change in enthalpy:

(2) $$\%wC=\frac{{dH}_{m}\times 100\%}{293}$$

The control was LDPE powder that was not treated with bacteria.

#### 2.4.3. Time-Domain Nuclear Magnetic Resonance

Samples of residual LDPE were collected after 2, 15, 21, 30, and 60 days of incubation with each bacterial culture and subjected to TD-NMR relaxometry. The analyses were conducted on a LexMar Global MagStation II (MS-2720) relaxometer (Haverhill, MA, USA), operated at a proton ^1^H resonance frequency of 20.05 MHz. The samples were automatically loaded into the probe and conditioned at 80 °C for 17 min*. Single pulse experiments were performed, and free induction decay (FID) signals were acquired at 0.2 µs/point. The FID signals were curve-fit with a 3-component model and the amplitudes of the crystalline, intermediate, and amorphous domains, as well as their respective spin–spin (T_2_) relaxation times were derived. The signals of the crystalline and intermediate components are represented by fast- and slow-decaying Gaussian functions, while an exponential function is used for the amorphous component. The function is as below:

(3) $$f(x)=A_{c}e^{-\frac{1}{2}{\left(\frac{x}{T_{c}}\right)}^{2}}+A_{i}e^{-\frac{1}{2}{\left(\frac{x}{T_{i}}\right)}^{2}}+A_{a}e^{-(\frac{x}{T_{a}})}$$

where, A_c_, A_i_, and A_a_ are the signal amplitudes of the crystalline, intermediate, and amorphous component, respectively. T_c_, T_i_, and T_a_ are the T_2_ relaxation times of these components in µs.

* Conditioning at 80 °C was longer than the standard method.

The NMR signal fitting was performed using OriginPro^®^ software (OriginPro 2021b SR2 9.8.5.212) using the nonlinear curve fit tool. The percentage of crystallinity (%C) and amorphous (%A) region were calculated as below:

(4) $$\%C=\frac{A_{c}}{\left(A_{c}+A_{i}+A_{a}\right)}\times 100\;and\;\%A=\frac{A_{a}}{\left(A_{c}+A_{i}+A_{a}\right)}\times 100$$

The control was LDPE powder that was not treated with bacteria.

### 2.5. Statistical Analysis

All samples were run in three biological triplicates. Data for crystallinity and amorphous content from TD-NMR, HT-SEC, and DSC were subjected to statistical analysis using Analysis ToolPak within Microsoft Excel (Version 2409). The significance level was set at *p* < 0.05 and triplicate samples were analyzed for each test.

## 3. Results

### 3.1. Bacteria Growth Using LDPE as the Sole Carbon Source

**Figure 1:**
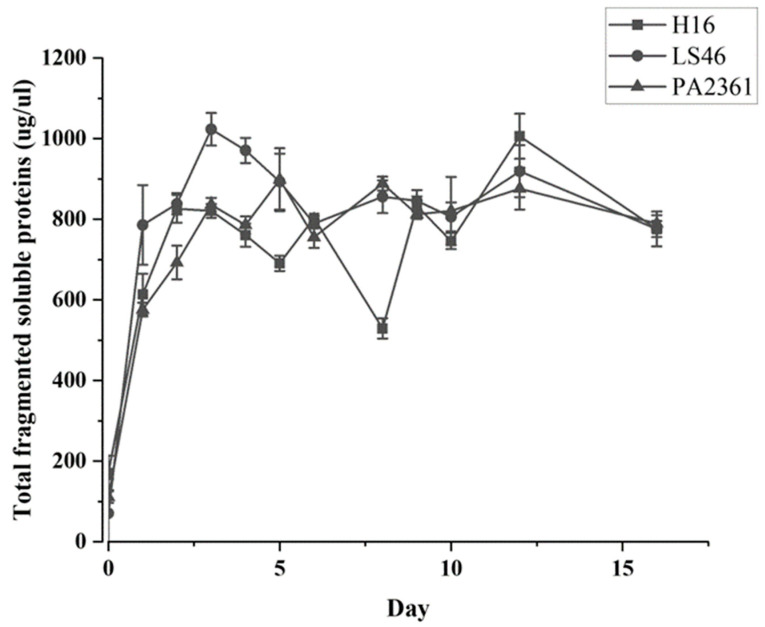
*C. necator* H16, *P. putida* LS46, and *P. chlororaphis* PA2361 growth curves in minimal media with no other carbon source besides LDPE (1% *w*/*v*). No growth was observed in the control and therefore it was not included in the graph.

The three bacteria’s growth on minimal medium with LDPE as the sole carbon source were monitored. The growth curves for each bacterium were generated based on the total amount of soluble fragmented proteins (Figure 1). All the strains show significant growth in the first three days of incubation before entering their stationary phase.

### 3.2. Degradation Products Produced during Bacteria Growth

Two definitive peaks of LDPE degradation products, identified as n-heptane (C7) and n-undecane (C11), were detected in the GC-FID chromatograms of all samples. Peaks that did not correspond with C7 and C11 alkanes, and did not match with the other standard peaks, were classified as ‘Unknown’ (Table 1). The peak intensities of the detected degradation products were calculated and presented as percent. Although the number and variety of products detected in the three LDPE-degrading bacterial cultures differ, the trend for each was similar. The amount of n-heptane (C7) was initially low, but then increased throughout the incubation period of the experiment (from 6.10% to 26.77%, 5.87% to 31.73%, and 9.12% to 27.44% for *C. necator* H16, *P. putida* LS46, and *P. chlororaphis* PA2361, respectively). The amount of n-heptane in the supernatant then decreased drastically at Day 60. On the other hand, the n-undecane increased throughout the first 21 days of growth, decreased at Day 30, and increased again at Day 60. The type of unknown degradation products ranged from C7 to C31; however, the exact identities of these products was not determined.

### 3.3. LDPE Weight Loss during Bacteria Growth

**Figure 2:**
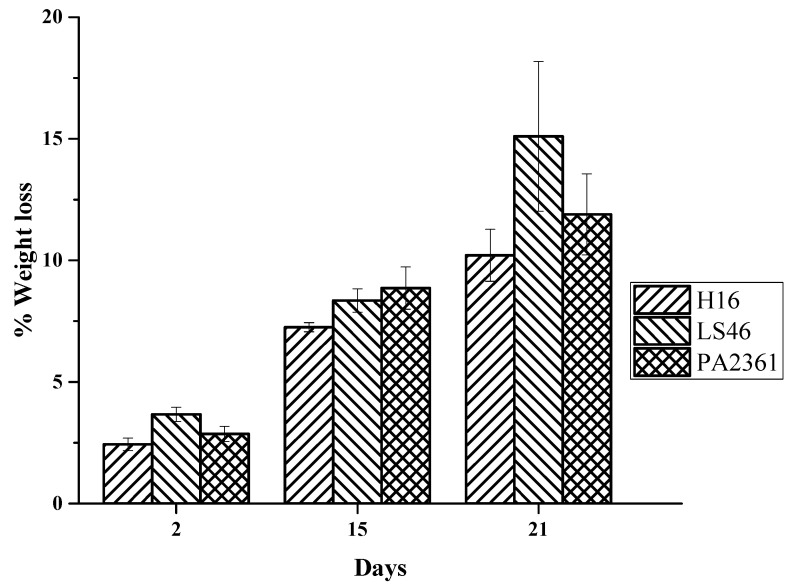
Percent weight loss of LDPE after exposure to *C. necator* H16 (H16), *P. putida LS46* (LS46), and *P. chlororaphis* PA2361 (PA2361) over 21 days of incubation.

The LDPE weight loss increases the longer the incubation period. The LDPE percent weight loss ranges from over 2% after 2 days of bacterial incubation to over 7% on Day 15. The % weight losses on Day 21 were 10 ± 1%, 15 ± 3%, and 12 ± 2% for *C. necator* H16, *P. putida* LS46, and *P. chlororaphis* PA2361, respectively (Figure 2).

### 3.4. LDPE Structural Changes after Incubation with Bacteria

#### 3.4.1. High-Temperature Size-Exclusion Chromatography (HT-SEC)

**Figure 3:**
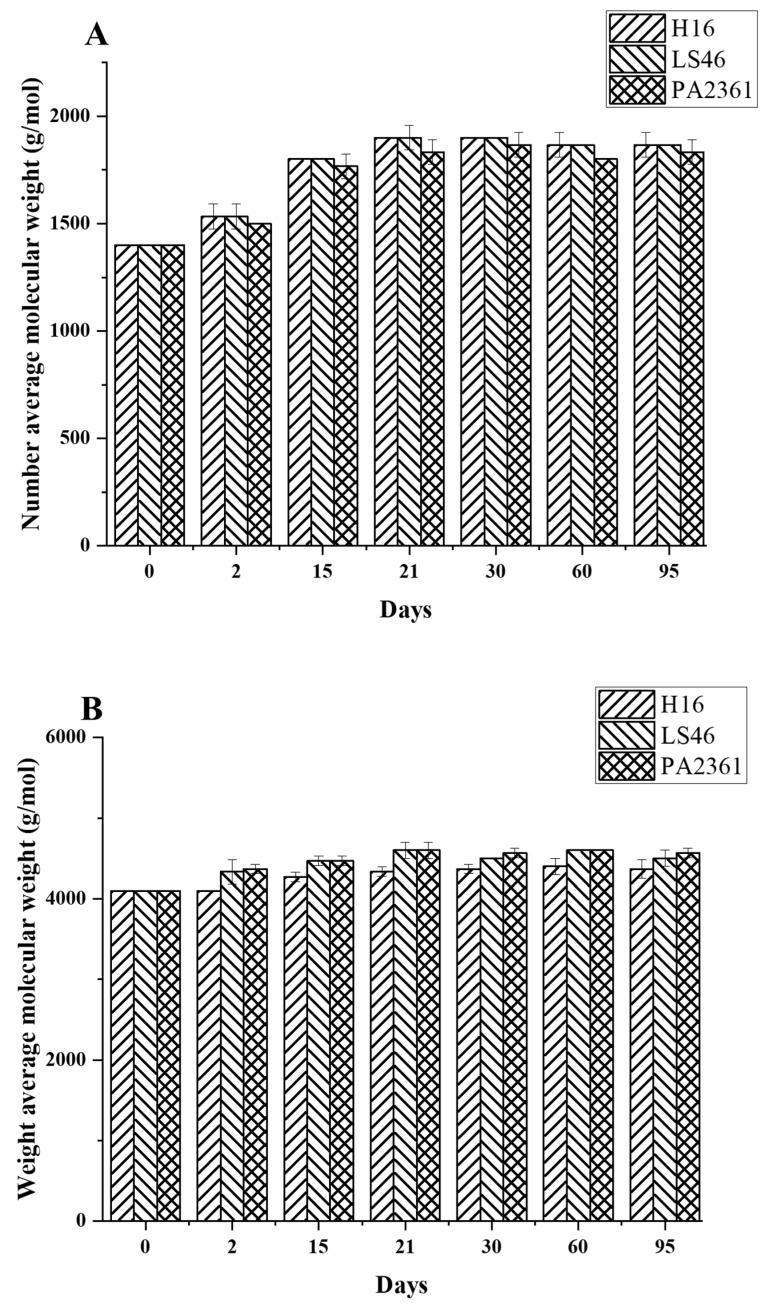

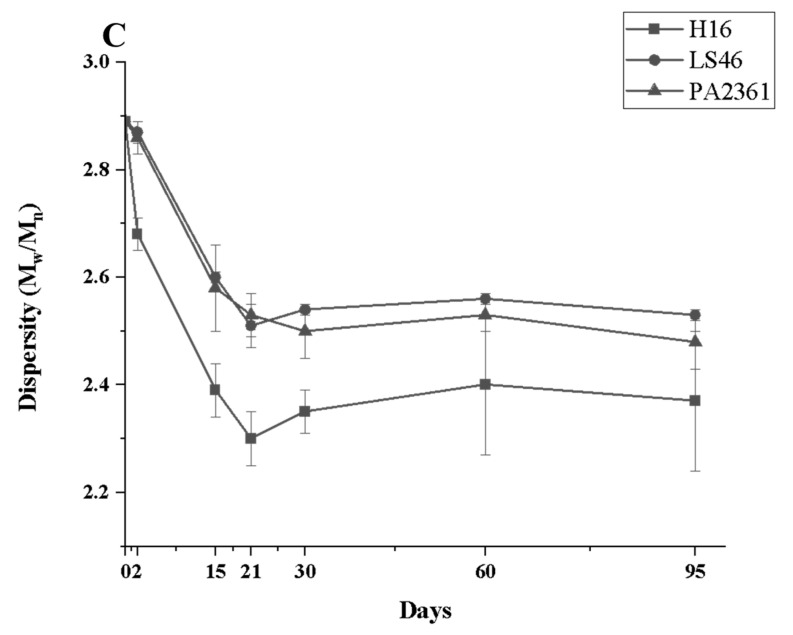
Changes in the structure of LDPE after incubation with three polymer-degrading bacteria. Changes in (**A**) the weight-average molar mass (M_w_), (**B**) the number-average molar mass (M_n_), and (**C**) the dispersity (M_w_/M_n_ = Đ) of LDPE powder after different incubation times with three bacteria, *C. necator* H16, *P. putida* LS46, and *P. chlororaphis* PA2361, analyzed by HT-SEC. LDPE standard is the LDPE that had no bacteria treatment.

**Figure 4:**
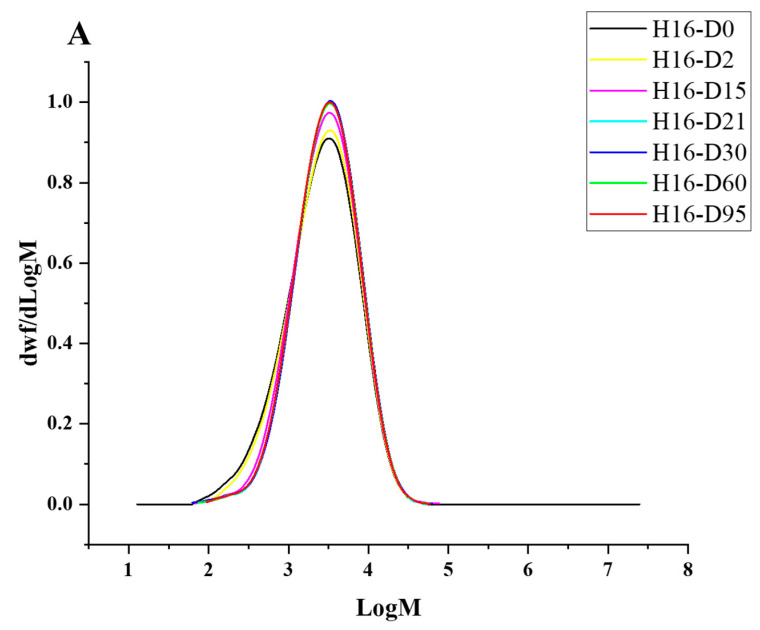

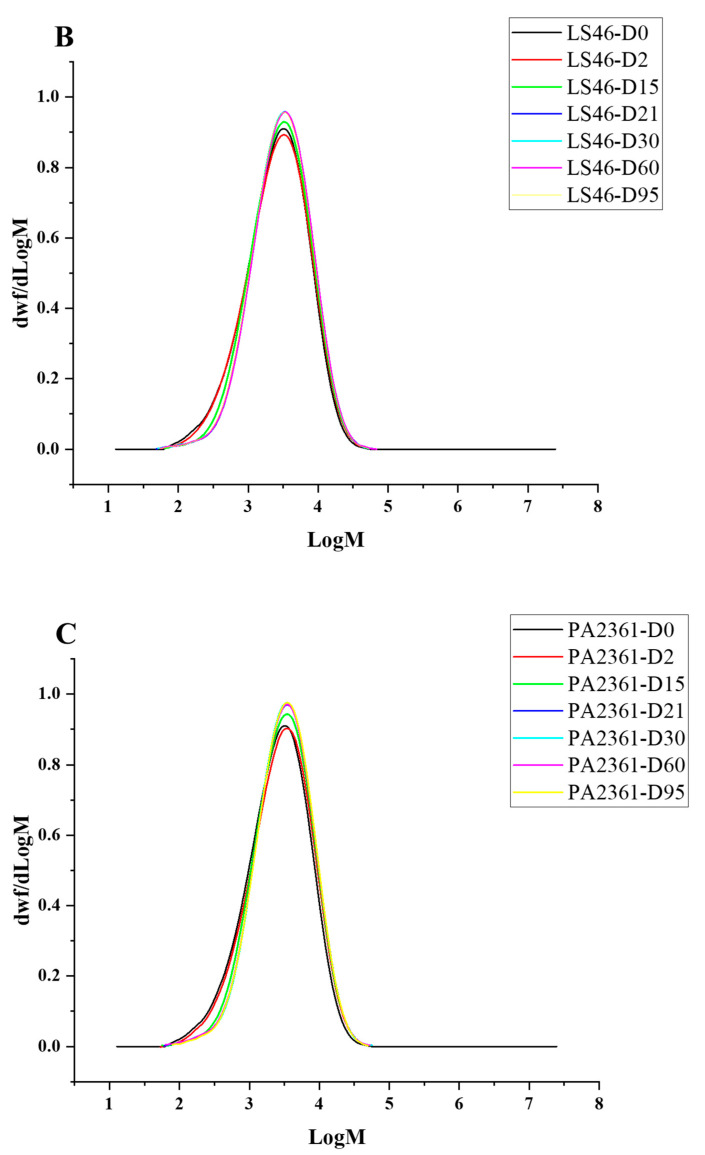
HT-SEC molar mass distributions of (**A**) *C. necator* H16-, (**B**) *P. putida* LS46-, and (**C**) *P. chlororaphis* PA2361-treated LDPE at different time points (Day 2, 15, 21, 30, 60, and 95). The black line represents the standard LDPE, which was not subjected to bacterial treatment.

Degradation to the LDPE structure was confirmed with HT-SEC. According to the results, both the weight-average molar mass (M_w_) and number-average molar mass (M_n_) increased as the incubation period length increased (Figure 3A,B). LDPE incubated with *P. putida* LS46 and *P. chlororaphis* PA2361 showed a significant increase in the M_w_ and M_n_ values after 2 days of bacterial incubation, but the dispersity (Đ) did not show significant changes until Day 15 (Figure 3C). In contrast, LDPE incubated with *C. necator* H16 did not display changes in the M_w_ after 2 days, but the M_n_ and Đ values showed significant changes compared to the control. In 2 days, the M_n_ value of LDPE incubated with *C. necator* H16 increased to 1530 g/mol, while the Đ decreased from 2.89 to 2.68. At Day 15, all three bacteria-treated samples showed similar trends in which the M_w_ and M_n_ values increased, and Đ decreased significantly. Additionally, the distribution of polymer molar mass was narrower as the length of the incubation period increased (Figure 4). The changes became insignificant after 21 days for all three bacteria and the polymer structure stayed approximately the same till Day 95.

#### 3.4.2. Differential Scanning Calorimetry (DSC)

DSC analyses indicated that the percent crystallinity remained constant at 40 to 41% after the first heating. However, the crystallinity content after the second heating increased slightly from 34% in the control to 36% at Day 60 for *C. necator* H16 and *P. putida* LS46, and 37% for *P. chlororaphis* PA2361 (Table 2). No significant changes in the wC content were observed between the control and all treated samples after the first heating cycle. There was, however, a slight increase in the wC content calculated based on the second heating cycle (34% to 36% for *C. necator* H16, and 34% to 37% for both *P. putida* LS46 and *P. chlororaphis* PA2361, respectively).

#### 3.4.3. Time-Domain Nuclear Magnetic Resonance (TD-NMR)

**Figure 5:**
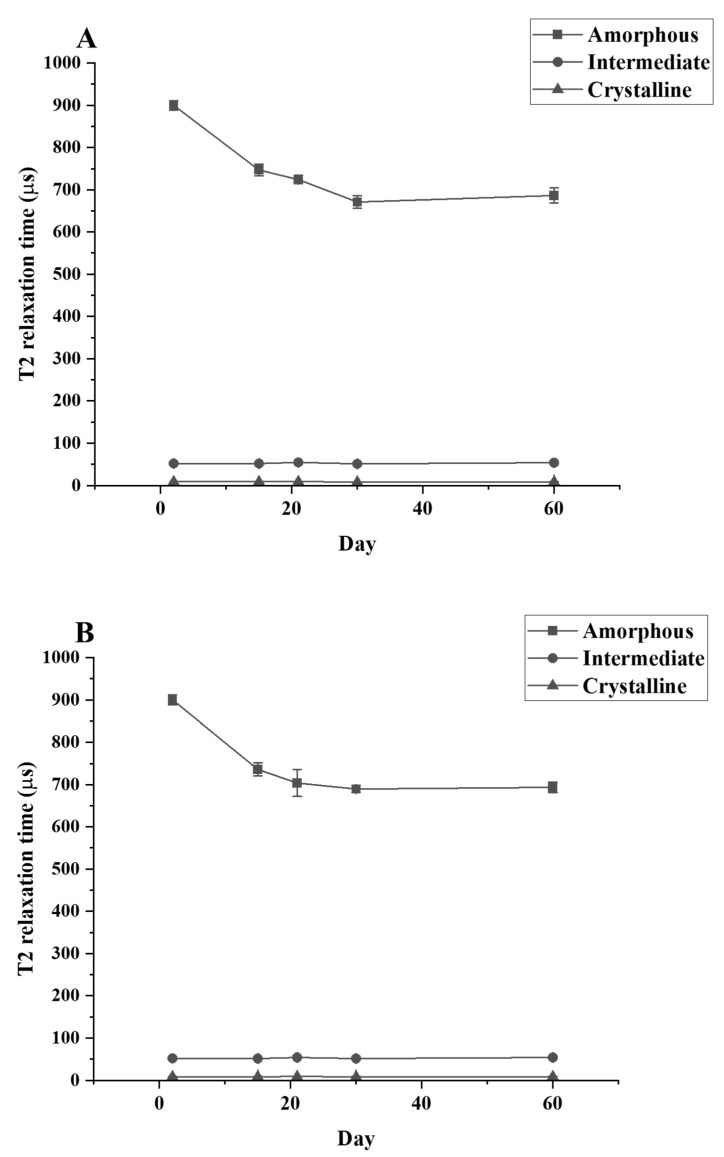

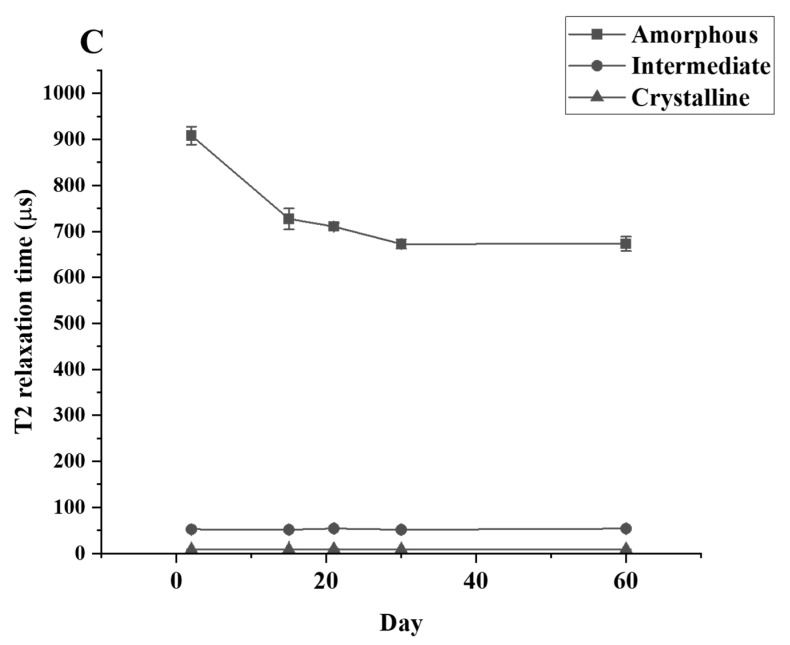
T_2_ relaxation times for LDPE samples after different periods of incubation with (**A**) *C. necator* H16, (**B**) *P. putida* LS46, and (**C**) *P. chlororaphis* PA2361, measured by TD-NMR.

The T2 relaxation time signal detected shows that only the amorphous region is changing during the culture period. The relaxation time of the amorphous region is significantly decreased between the control and Day 30 samples while the relaxation time for the crystallinity and the semi-crystalline regions remain unchanged (Figure 5). The data from TD-NMR also allow the quantification of the amorphous, crystallinity, and semi-crystallinity content. The amorphous content of LDPE decreased, while the crystallinity content increased in the LDPE incubated with each culture. During the first 30 days of incubation, the amorphous content of LDPE decreased significantly from 47.62% to 39.59%, 40.05%, and 39.92% for *C. necator* H16, *P. putida* LS46, and *P. chlororaphis* PA2361, respectively (Table 3). The changes in the percentage of amorphous regions of the polymer were not significantly different between Day 30 and Day 60 for all samples. In contrast, the crystallinity content increased significantly during the first 30 days of incubation, from 45.89% in the control to 50.92%, 50.46%, and 50.49% in LDPE incubated with *C. necator* H16, *P. putida* LS46, and *P. chlororaphis* PA2361, respectively. Similar to the amorphous content changes, the crystallinity contents were insignificantly different between Day 30 and Day 60 for all samples.

## 4. Discussion

The three species of bacteria were cultured in minimal PE media with LDPE as the sole carbon source, and growth curves were used to determine the cell mass increase (Figure 1). The presence of LDPE powder in the media causes interference in the OD_600_ readings; thus, the growth curves were based on the total soluble protein extracted from cell pellets after various times of incubation. The three bacteria grew rapidly during the first 2 days of incubation. As the bacteria grew on LDPE as a sole carbon source, it was assumed that the bacteria secreted enzymes that degraded the LDPE into smaller fragments that were assimilated and catabolized by the bacteria. Indeed, fragments generated by LDPE hydrolysis were detected in the supernatants of the bacterial cultures. The degradation products detected were identified as n-heptane (C7 alkane), undecane (C11 alkane), and a number of unknown products.

These results are consistent with previously reported data [15,24,25]. The unknown products could potentially be aldehydes, ketones, or carboxylic acids compounds generated by oxidation of the LDPE polymer chains. The amount of each type of product is slightly different for each bacterial species, but they all had similar trends. The amounts and types of fragments suggest that the enzymes from the three bacteria may share similar substrate preferences, but with different activities. The result of GC-FID indicates that there are smaller linear alkane fragments and other variations present in the supernatant due to the bacterial enzymes’ activity. The lengths of degradation products do not correspond with typical short chain lengths of LDPE (four to five carbon atoms), suggesting the polymer chains are split in linear parts of the chain backbone rather than at tertiary carbon atoms of branching points. This indicates quite a different mechanism of enzymatic degradation compared to classical photooxidation. Unfortunately, the exact identity of the degradation products is not identified in this study. Identification of these products is essential for further investigation of enzymatic preferred substrates and designing microbial consortium to maximize LDPE degradation. For the purposes of this study, the presence of degradation products in the supernatant plus the decrease in LDPE weight and the increase in bacteria growth indicate that the bacteria are utilizing the LDPE for their growth.

The amount of LDPE weight loss in the current study was not as high as previously reported [14]. This could be due to the difference in the media used. Since *C. necator* H16 and *P. putida* LS46 are known for producing polyhydroxyalkanoate (PHA) under stress conditions, the previous study used Ramsay medium, which has a lower nitrogen and phosphorus content compared to the one used here to induce PHA production. Additionally, linear n-alkanes can potentially be used as a substrate for PHA production. Thus, the amount of LDPE used by Montazer et al. [14] was higher, and the bacteria used the LDPE substrate for both growth and PHA synthesis. Our data suggest that LDPE degradation can be coupled with another n-alkane-consuming process to increase the degradation of LDPE, as well as potential secondary products being produced from biodegradation of LDPE.

Fragmentation of the LDPE by enzymatic degradation would result in changes in the LDPE structure, which was analyzed using different methods. High-Temperature Size-Exclusion Chromatography (HT-SEC) detected increases in both M_w_ and M_n_ values, suggesting that the LDPE chains were cleaved, especially at the lower molecular weight fractions. HT-SEC confirmed changes in LDPE molar mass distribution by revealing small, but significant, shifts in the dispersity, and increases in both the Mw and Mn values. Similar changes in M_n_ and M_w_ values in biodegraded HDPE were also detected using GPC, suggesting degradation at the lower molecular weight fragments of the polymer [17,19,25]. Those low molecular weight fragments of the LDPE belong to the amorphous content of the polymer. As a result, LDPE degradation occurs by preferential degradation of LDPE fractions with the lowest molar mass (waxes) that are more accessible to the bacterial enzymes. However, although LDPE degradation was confirmed after incubation with the bacterial cultures, the extent of degradation was limited to the amorphous regions of the polymer in the first 21 days of incubation and did not continue after that. The availability of the amorphous regions might be the reason for the limited degradation seen here.

Differential Scanning Calorimetry (DSC) detected changes in the percentage of crystallinity (%wC) of the polymer. The insignificant changes between samples and control in the first heating represent the crystallinity amount of the LDPE powder after being treated with bacteria. However, the first heating possibly removed impurities in the LDPE powder, which led to a decrease in the %wC when comparing the first and second heating. Enzymatic degradation of the amorphous regions of the LDPE could remove some of the alkane branches in the LDPE structure, which would impact the refolding of the LDPE after the first heat of enthalpy test, hence the difference in %wC detected in the second heat of enthalpy test. Therefore, the data here suggest that there were changes in the LDPE structure, but the crystalline regions were unaffected. These results contrast with the report by Albertsson et al. [26], which proposed that the microorganism *Arthobacter paraffineus* attacks both the crystallinity and amorphous part of the LDPE polymer. This conclusion was based on the change in M_w_, M_n_, and the crystallinity content measured by DSC and X-ray Diffraction (XRD) that was previously published [26]. However, the LDPE used there was modified to contain starch, a pro-oxidant, and pre-treated at 100 °C. The presence of a pro-oxidant and pre-treatment of LDPE would increase the LDPE degradation by introducing carboxylic groups into the structure [19]. These treatments would also loosen up the LDPE structure, causing a decrease in the crystallinity of the polymer. Thus, further testing is required to confirm which regions of the LDPE is being degraded during biodegradation.

A reduction in the amount amorphous regions in the polymer while the percentage of crystallinity remained constant was demonstrated experimentally via TD-NMR relaxometry. This method allows measurement of the hydrogen mobility within the polymer samples. This mobility is represented by “T_2_ relaxation”. In amorphous regions, the polyethylene chains are less tightly packed, and thus would display a higher T_2_ relaxation rate. The higher amorphous content, the greater the mobility, and therefore a higher relaxation rate. In contrast, the tight arrangement of the crystalline regions restricts hydrogen vibration, which would lead to a lower T_2_ relaxation rate. Figure 5 shows that the crystallinity content remains constant while the amorphous region decreased during the 21 days of bacteria incubation. The constant amount of crystallinity is correlated with the lack of change in the %wC detected by DSC after the first heating, confirming that there were no changes in the crystallinity content before and after bacteria treatment.

Using Equation (4), the TD-NMR data enabled the calculation of percentages of the amorphous and crystalline regions of untreated and treated LDPE, and revealed that the amount of amorphous and crystalline regions changed throughout the 21 days of the bacteria incubation period. It is noted that the crystalline content calculated here is different from that calculated by the DSC method, especially the %wC calculated in the second heating cycle. The DSC method reveals the bulk crystallinity content in the LDPE powder and is not as sensitive when it comes to the amorphous content. On the other hand, TD-NMR measures signals from the entire hydrogen population of the polymer, and fractional contents are calculated using Equation (3). Thus, TD-NMR is more sensitive to the amorphous content since it measures hydrogen mobility within the structure. The decrease in T_2_ relaxation rate yields a more accurate change in the % amorphous content. TD-NMR allows calculation of the distribution of each content in relation to one another, which explains the increase in % crystallinity content seen in Table 3. By using this method, one can calculate the weight of amorphous and crystallinity distributed in a specific sample before and after biodegradation. In this experiment, only changes in the amorphous weight were observed, while the weight of the crystallinity portion remained unchanged. The results suggests that polymers with higher amorphous content would be easier to biodegrade. Furthermore, methods to increase amorphous regions such as pre-treatment with UV light or chemicals would enhance the biodegradation process.

In conclusion, the three bacteria here show the ability to degrade LDPE and use degradation products for growth. Even though each bacteria strain has different efficiency in degrading LDPE, the three bacteria show similar trends in biodegradation mechanism and that the degradation of LDPE can be seen during the first 21 to 30 days. Multiple evidence from DSC and HT-SEC analyses has indicated that the LDPE crystallinity content remains constant throughout the biodegradation process. The TD-NMR data provide strong evidence that the three bacteria only attack the amorphous region of the LDPE, suggesting that the resistance of synthetic polymers to biodegradation is related to the percentage of crystallinity of the polymer.

## 5. Conclusions

The amorphous regions of LDPE are less tightly packed compared to the crystalline regions, and contain extruding branches, which prevent folding into highly ordered, tightly packed crystalline structures. Our data suggest that the spatial organization of the amorphous regions allows bacterial enzymes to interact with and cleave these regions of the polymer first. Once the available amorphous regions are degraded, the polymer tends to refold in a more compact manner, preventing further amorphous region exposure. The reduction in accessible amorphous regions limits the bacterial enzymes’ activity, which increases the resistance of the LDPE to biodegradation. The results of this study point to the degree of crystallinity as the main limitation in the biodegradation of LDPE. Further investigation of the relationship between LDPE structure and polymer-degrading enzymes may lead to strategies to decrease the percentage of crystallinity, increasing the extent of LDPE biodegradation. Thus, understanding how polymer structure impacts the ability of polymer-degrading enzymes to breakdown plastic materials may be the key to developing engineered solutions to plastic pollution.

## Figures and Tables

**Table 1:** Distribution percentage of the hydrolysis products from the supernatant of (A) *C. necator* H16, (B) *P. putida* LS46, and (C) *P. chlororaphis* PA2361 cultures. The values were calculated by taking the average area of each peak. Degradation products were identified by matching the peaks with those in the C7–C40 linear alkane standard. ‘Unknown’ refers to those peaks that do not match the standard linear alkane peaks. The controls show no detectable peaks besides the chloroform and internal standard.

**(A)**	**Day**				
	**2**	**15**	**21**	**30**	**60**
C7	6.10%	10.93%	10.83%	26.77%	2.58%
C11	19.66%	67.03%	74.16%	61.55%	92.11%
Unknown	74.24%	22.04%	15.01%	11.68%	5.31%
**(B)**	**Day**				
	**2**	**15**	**21**	**30**	**60**
C7	5.87%	8.83%	10.05%	31.73%	11.56%
C11	21.46%	67.78%	75.78%	57.07%	71.74%
Unknown	72.67%	23.39%	14.17%	11.20%	16.70%
**(C)**	**Day**				
	**2**	**15**	**21**	**30**	**60**
C7	9.12%	9.93%	10.88%	27.44%	10.86%
C11	63.32%	65.13%	72.96%	63.68%	65.13%
Unknown	27.56%	24.94%	16.16%	8.88%	24.01%

**Table 2:** Melting temperature (T_m_) and the crystallinity percentage (wC%) in LDPE before and after incubation with *C. necator* H16, *P. putida* LS46, and *P. chlororaphis* PA2361. Control, LDPE that was not incubated with any bacterial culture. dH_m_ is the normalized change in enthalpy. D# indicates the number of days of incubation with each bacterial culture. Data are presented as value ± standard deviation.

	First Heating			Second Heating		
	T_m_ (°C)	dH_m_ (J/g)	wC (%)	T_m_ (°C)	dH_m_ (J/g)	wC (%)
Control	102.0 ± 0.0	119.0 ± 1.4	40.5 ± 0.7	104.0 ± 0.0	101.0 ± 1.4	34.5 ± 0.7
H16-D2	103.7 ± 0.6	118.7 ± 2.3	40.7 ± 0.6	104.0 ± 0.0	100.0 ± 1.0	34.0 ± 0.0
H16-D15	103.3 ± 0.6	116.0 ± 1.0	39.7 ± 0.6	103.7 ± 0.6	103.3 ± 2.1	35.0 ± 1.0
H16-D21	103.7 ± 0.6	120.7 ± 2.1	41.3 ± 0.6	104.0 ± 0.0	104.7 ± 1.2	35.3 ± 0.6
H16-D30	104.0 ± 0.0	122.3 ± 1.2	41.7 ± 0.6	104.0 ± 0.0	106.0 ± 1.7	36.3 ± 1.2
H16-D60	103.7 ± 0.6	119.0 ± 1.0	40.7 ± 0.66	104.0 ± 0.0	106.7 ± 1.2	36.0 ± 1.0
LS46-D2	104.7 ± 0.6	117.0 ± 1.7	39.7 ± 0.6	104.0 ± 0.0	97.7 ± 3.5	33.0 ± 1.0
LS46-D15	103.0 ± 0.0	118.3 ± 0.6	40.3 ± 0.6	103.7 ± 0.6	103.3 ± 1.5	35.3 ± 0.6
LS46-D21	103.3 ± 0.6	123.7 ± 1.2	41.7 ± 0.6	104.0 ± 0.0	107.3 ± 2.1	36.7 ± 0.6
LS46-D30	103.7 ± 0.6	123.7 ± 1.5	41.7 ± 0.6	104.0 ± 0.0	109.3 ± 0.6	37.3 ± 0.6
LS46-D60	103.3 ± 0.6	118.7 ± 0.6	40.7 ± 0.6	104.0 ± 0.0	106.3 ± 0.6	36.3 ± 0.6
PA2361-D2	104.0 ± 0.0	117.3 ± 0.6	40.0 ± 0.0	104.0 ± 0.0	98.3 ± 3.2	33.7 ± 1.2
PA2361-D15	103.7 ± 0.6	118.3 ± 1.5	40.3 ± 0.6	104.0 ± 0.0	102.3 ± 1.5	34.7 ± 0.6
PA2361-D21	103.3 ± 0.6	125.3 ± 0.6	43.0 ± 0.0	104.0 ± 0.0	108.0 ± 0.0	37.0 ± 0.0
PA2361-D30	103.0 ± 0.0	119.3 ± 6.4	40.7 ± 2.3	104.0 ± 0.0	104.0 ± 5.2	35.7 ± 2.3
PA2361-D60	104.0 ± 1.0	120.3 ± 1.5	41.3 ± 0.6	104.0 ± 0.0	107.3 ± 0.6	37.0 ± 0.0

**Table 3:** Percent amorphous (% Amorph) and crystalline regions (% Crystal) of LDPE after different periods of incubation with *C. necator* H16, *P. putida* LS46, and *P. chlororaphis* PA2361, measured by TD-NMR. The experimental control was LDPE incubated without bacterial culture, indicated as D0. D# indicates the number of days of incubation with each bacterial culture.

		D0	D2	D15	D21	D30	D60
H16	% Amorph	47.62	45.36 ± 0.25	42.03 ± 1.27	41.49 ± 0.40	39.59 ± 0.36	40.31 ± 0.55
	% Crystal	45.89	45.94 ± 0.28	48.51 ± 1.33	49.22 ± 0.36	50.92 ± 0.21	50.53 ± 0.50
LS46	% Amorph	47.62	45.26 ± 0.61	40.74 ± 0.90	41.15 ± 0.98	40.05 ± 0.26	40.32 ± 0.27
	% Crystal	45.89	46.00 ± 0.55	49.94 ± 0.64	49.55 ± 0.92	50.46 ± 0.25	50.59 ± 0.21
PA2361	% Amorph	47.62	45.17 ± 0.93	40.79 ± 1.08	41.50 ± 0.61	39.92 ± 0.65	39.55 ± 0.59
	% Crystal	45.89	46.10 ± 0.85	49.72 ± 1.00	49.17 ± 0.53	50.49 ± 0.81	51.29 ± 0.54

## Data Availability

The raw data supporting the conclusions of this article will be made available by the authors on request.
